# Effect of Forging Parameters on Low Cycle Fatigue Behaviour of Al/Basalt Short Fiber Metal Matrix Composites

**DOI:** 10.1155/2013/250513

**Published:** 2013-11-05

**Authors:** R. Karthigeyan, G. Ranganath

**Affiliations:** Adhiyamaan College of Engineering, Hosur 635 109, Tamil Nadu, India

## Abstract

This paper deals with metal matrix composites (MMCs) of Al 7075 alloy containing different weight percentage (2.5, 5, 7.5, and 10) basalt short fiber reinforcement and unreinforced matrix alloy. The samples were produced by the permanent stir casting technique. The casting ingots were cut into blanks to be forged in single stage and double stage, using MN press and graphite-based lubricant. The microstructures and fatigue properties of the matrix alloy and MMC samples were investigated in the as cast state and in the single and double stage forging operations. The microstructure results showed that the forged sample had a uniform distribution of the basalt short fiber throughout the specimens. Evaluation of the fatigue properties showed that the forged samples had higher values than those of the as cast counterparts. After forging, the enhancement of the fatigue strength of the matrix alloy was so significant and high in the case of 2.5 and 5.0 wt. percentage basalt short fiber reinforced MMC, and there was no enhancement in 7.5 and 10 weight percentages short fiber reinforced MMCs. The fracture damage was mainly due to decohesion at the matrix-fiber interface.

## 1. Introduction

Metal matrix composites (MMCs) with better mechanical strength are under development in order to introduce structural components for automotive and aerospace applications, by reducing drastically the weight and increasing the specific strength [1]. But the particular microstructural changes in MMCs induce stiffness in the direction of fiber to poor transverse direction (transverse direction means perpendicular direction to basalt fiber) due to the weak interface between the matrix and the fiber. The severity of this problem is higher in the case of short fiber reinforced MMCs. On the other hand, as the stiffness and strength are increased, a substantial decrease in ductility is found. To overcome these problems, many researchers devoted themselves to the study of the effect of mechanical working such as extrusion [2], rolling [3], and forging [4] on MMCs, and they have shown that some improvements in strength and ductility can be observed with the application of short fiber reinforced Al MMCs [5]. However, if the forging process parameters are not adequately controlled, the composite shows a nonhomogeneous fiber orientation and then a reduction of the mechanical properties [6–8].

The fatigue resistance of short fiber reinforced MMCs depends on various factors such as the type and weight percentage of reinforcements, fiber aspect ratio, fiber and matrix interfacial bonding, the presence of inclusions or defects that arise from processing, and testing environment [9, 10]. The shortfibre reinforcement also leads to poor ductility and low fracture toughness [11]. To be able to fully use the properties of this kind of materials, a knowledge of fatigue behaviour and an improved understanding of strain-controlled low-cycle fatigue (LCF) damage tolerance characteristics in these materials are outstandingly required [12, 13]. Hence, the objective of this work is to investigate the effect of forging parameters on microstructure and fatigue behaviour of a basalt short fiber reinforced Al MMCs at room temperature. The basalt short fiber was varied from 2.5 to 10 percentages in an interval of 2.5 weight. percentage to study the fracture surface damage associated with particle fracture and interfacial bonding strength.

## 2. Experimental Studies

### 2.1. Materials

The matrix alloy used in the present investigation was Al alloy, which had Cu coated basalt short fiber of diameter 3–5 *μ*m as reinforcement and chemical composition as shown in Table 1. Fibers in roving form were bundled and cut into short fibers of uniform length of about 1 to 2 mm by constant-length cutter. The short basalt fiber was cleansed in distilled water and dried at 90°C under force convection to remove impurity and dirt.

### 2.2. Composite Preparation

The composites were prepared by adding 0, 2.5, 5, 7.5, and 10 weight. percentage of basalt short fiber using liquid metallurgy technique. The basalt short fibers were introduced into the molten metal pool through a vortex created in the melt by the use of an alumina-coated stainless steel stirrer. The coating of alumina on the stirrer is essential to prevent the migration of ferrous ions from the stirrer material into the molten metal. The stirrer was rotated at 550 rpm, and the depth of immersion of the stirrer was about two-thirds of the depth of the molten metal. The preheated (500°C) basalt short fibers were added into the vortex of the liquid melt which was degassed using pure nitrogen gas for about 3 to 4 min. The resulting mixture was tilt and poured into preheated permanent moulds (400°C).

### 2.3. Forging and Heat Treatment Operations

**Figure 1:**
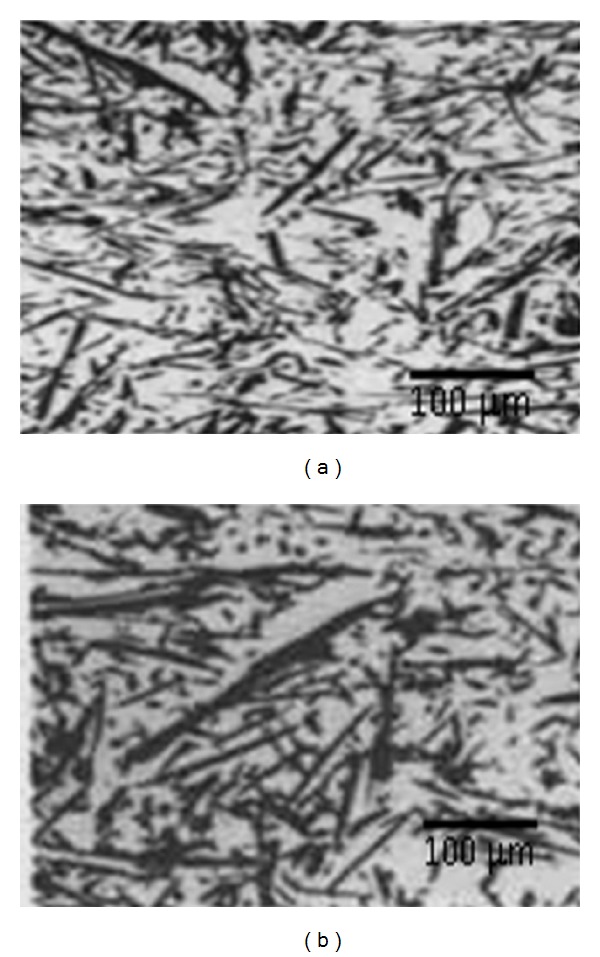
The microstructure of the Al/10% basalt short fiber MMCs (a) as cast and (b) in forged conditions.

Forging was carried out in an open die in both single and double stages, using a 20 MN press and a graphite-based lubricant. The temperature of the composites at the beginning of the process was about 500°C, while at the end of the forging it was 425°C. The forging parameters were temperature of the die 400°C; initial billet height 75 mm, final billet height 25 mm, and deformation ratio 3 : 1; average engineering strain rate 0.1 s-1 as per Hong et al. [13]. The flat specimens (Figure 1) tested in situ were machined using an electron-erosion technique. The heat treatment (T6) used to achieve these conditions was a solution treatment of 10 hours at a temperature 532°C followed by water quenching on hot water at 80°C aged at room temperature for 12 hrs followed by ageing at 175°C for 3 hours. Composites specimens were prepared by standard mechanical polishing techniques and etched with Keller's reagent and microstructural examination by using optical microscope.

### 2.4. Low Cycle Fatigue Test

**Figure 2:**
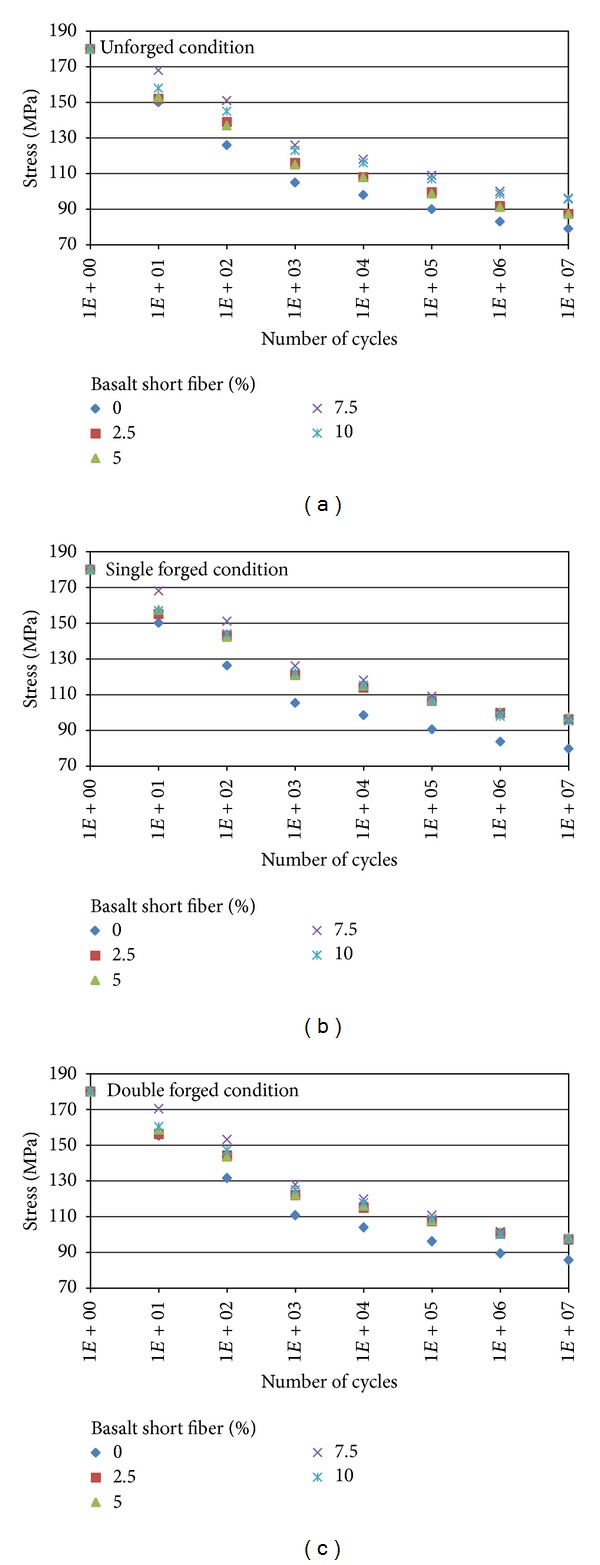
Wöhler curves for Al and Al/basalt short fiber composites for (a) unforged, (b) first stage forging, and (c) second stage forging.

**Figure 3:**
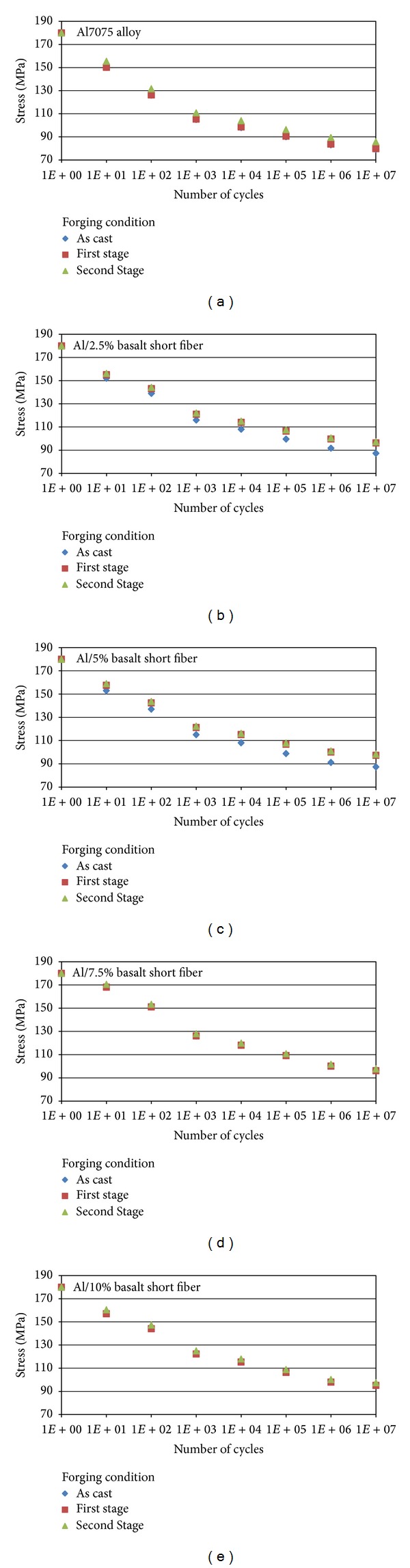
Wöhler curves for as cast, first stage forging, and second stage forging for Al, Al/2.5%, Al/5%, Al/7.5%, and Al/10% basalt short fiber MMCs.

After heat treatment, total-strain controlled fatigue tests were carried out with total-strain amplitudes ranging from 0.001 to 0.01 in servohydraulic universal testing machines. Strain was measured by clip-on extensometer attached directly to the gauge length at room temperature. The stress level corresponding to the LCF (compression and tension) was fixed nominal stress amplitude of 180 MPa with a constant load rate equal to 125 N/s. The fatigue tests have been carried out in load control (in a fully reversed push-pull mode) with hydraulic test machine, and a comparison of the stress versus cycles to failure behaviour of the unforged, single, and double forged specimens belongs to both Al7075 matrix alloy and Al/basalt composites as shown in Figures 2 and 3, respectively.

## 3. Results and Discussion

### 3.1. Microstructure Studies

Figure 1 shows the optical microstructure of the Al/10 percentage basalt short fiber composites in the as cast and in the forged conditions. The forged microstructure of the Al/10 percentage basalt short fiber composites exhibited some preferential alignment of basalt fiber perpendicular to the forging direction. The basalt short fibers tended to align themselves in such a way that the smallest dimension was parallel to the forging direction.

### 3.2. Fatigue Resistance

Figure 2 explains an increase in the content of basalt short fiber that resulted in enhanced fatigue resistance of the composites independent of orientation.

However, the magnitude of enhancement fatigue resistance is higher at lower weight percentage of basalt short fiber (0–5 wt. percentage) but no significant change can be seen at a higher percentage (7.5 and 10 wt. percentage). Several studies have shown that the increasing weight percentage of reinforcement results in enhanced fatigue resistance [14]. Since most of the load in the composites is carried by the high modulus basalt fiber, the composite undergoes a lower average strain than the matrix alloy. Thus, the fatigue lives of basalt short fiber reinforced MMCs are generally longer than those of matrix alloys. These improvements are most pronounced at lower stresses in the high-cycle fatigue regime, while at high stress, the differences between reinforced and unreinforced materials are reduced.

The change in fatigue resistance as a function of the forging condition is given in Figure 3 for the matrix alloy and composites specimens. The curves show that all the specimens exhibit a common behaviour with a number of cycles. The fatigue strength in forged condition specimen (single stage and double stage) shows that there is no significant change in matrix alloy, but 2.5 and 5 percentage basalt short fiber reinforced MMCs shows significant changes in their behaviour as shown in Figure 3. On the other hand, there is no significant effect of forging on fatigue resistance of 7.5 percentage and 10 percentage reinforcement. The dominant factors controlling the elastic modulus in MMCs are the weight percentage of the reinforcement, the aspect ratio of reinforcement, and the load-transfer capability of the interface, but the presence of porosity and microcrack would reduce the modulus [15]. The results presented by Lee et al. [16] and by Lloyd [17] show that the application of deformation to the composite materials reduces the elastic modulus. The mechanism of the change is complicated; however, fiber cracking, interface debonding, and, possibly, dislocation structure change under external strain were all considered to be responsible for the reduction in modulus. In the recent study, the effect of the deformation on the elastic modulus is not expected to be observable. This is probably due to the reduction in porosity by forging, as the reduction in modulus induced by deformation may be compensated by the increase resulting from the reduced porosity in the samples of this study.

### 3.3. Fracture Studies

**Figure 4:**
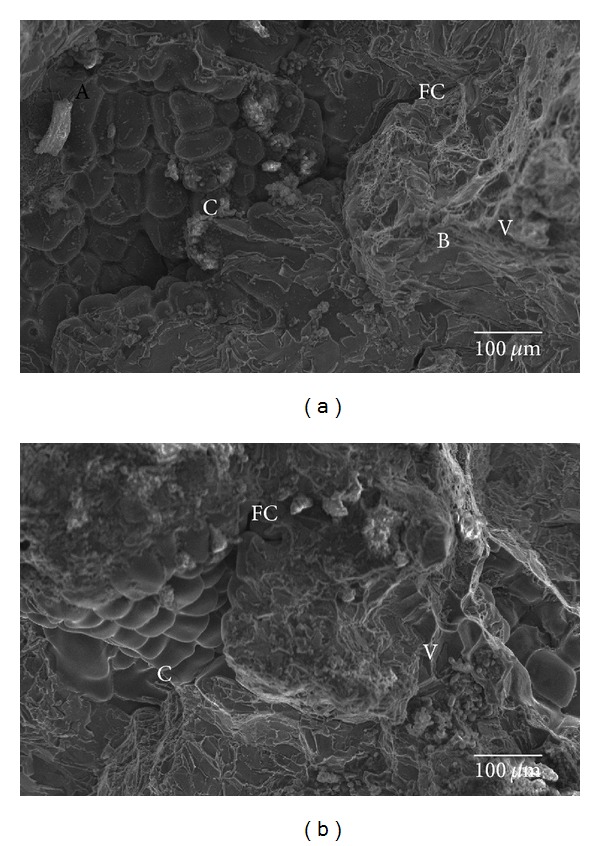
Fracture surface of (a) unforged and (b) forged condition Al7075 alloy specimen after fatigue. C-main crack, FC-fatigue crack, and V-void in matrix alloy.

**Figure 5:**
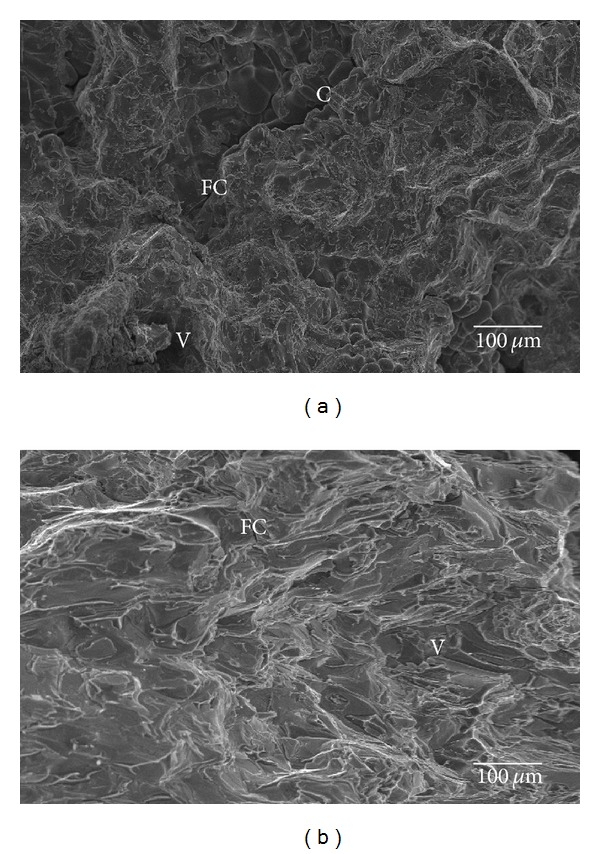
Fracture surface of (a) unforged and (b) forged condition Al 7075/5 weight percentage of basalt short fiber MMCs after fatigue test. C-main crack, FC-fatigue crack, and V-void in matrix alloy.

The typical fractured surface of Al 7075 alloy and Al/5 weight percentage basalt short fiber composites (both as cast and two-stage forged condition) after fatigue test are shown in Figures 4 and 5, respectively.  Figure 4(a) shows after crack initiation at pores, two distinct fracture morphologies were observed and marked the same “A” (ductile fracture) and “B” (brittle fracture) and fatigue crack (FC) propagated between these two regions. The region “A” (ductile fracture) dominates more than the region “B,” (brittle fracture) and the fatigue crack is formed in the severe damage region.  Figure 4(b) shows fracture surface of both of the two stages forging.

Al 7075 alloy after fatigue failure contains two regions, “A” (ductile failure) and “B” (brittle failure). The region B is more dominante than the region “A” which due to foringing reduces the porosity and refines the grain boundaries in the matrix alloy. Similar to unforged specimen, the crack (C) and fatigue crack(FC) are continuous and formed in the severe damage region.

Figures 5(a) and 5(b) show the fracture surface of as cast and forged conditions of Al/5percentage basalt fiber reinforced composites specimens, respectively. Unlike Al matrix alloy, these do not show any two clear distinct regions in both as cast condition and forged condition. Cavaliere [1] noted that as the interspacing decreased, the degree of constraint due to triaxiality of stress increased, so striation formation was hindered, and the dominant damage mechanism was changed to void formation. After stable crack propagation, a fast fracture region was typically observed. Because of the high-crack velocity associated with this portion of the fracture surface, large-scale fracture took place.

The fracture surfaces also displayed the presence of few cracks (Figure 5(b)), probably originated from casting defects or during cooling from the fabrication. This is in accordance with the increased density of the composite after forging. Because of reduction in porosity, the ductility of composites increases, and it leads to the large elongated dimples, tear ridges, and shear bands which are shown in the fracture surface. Moreover, both the unforged and forged MMC materials exhibited similar interface debonding in fracture, which created additional secondary microcracks due to continued fatigue cycling. Numerous voids were formed ahead of the crack tip, and the microcracks intersected with other nearby microcracks [18].

## 4. Conclusion

In the present work, the effects of basalt short fiber content and the forging process on the fatigue properties of Al 7075/basalt short fiber MMCs are studied, and the conclusions are drawn from the experimental observations as follows.

1. The microstructures of the as cast and forged Al/basalt short fiber MMCs exhibit uniform distribution of basalt short fiber, and porosity content in forged specimen is substantially reduced.
2. The matrix alloy and MMCs behave more like a monolithic material, since the homogeneous spatial distribution of the basalt short fiber enables efficient load transfer from the matrix to the reinforcement fiber without producing stress concentration sufficient to initiate fatigue cracks.
3. With increasing basalt fiber content, the fatigue value has been increasing up to 7.5 percent in the as cast, single, and double stage forged specimens, and it has been found that there is no significant change when more than 7.5 weight percentage of short basalt fiber is added.
4. SEM analyses of the fracture surfaces showed the broken particles, surrounded by ductile region, and decohesion at the matrix and fiber interfaces. The tear ridges in the MMCs can be explained with the high local plastic constraints induced by fiber cluster.

## Figures and Tables

**Table 1:** Chemical composition of Al 7075 alloy-weight percentage.

Element	Si	Fe	Cu	Mn	Mg	Cr	Zn	Ti	Al
Percentage	0.4	0.5	1.6	0.3	2.5	0.15	5.5	0.2	Bal

## References

[1] Cavaliere P (2004). Isothermal forging of AA2618 reinforced with 20% of alumina particles. *Composites A*.

[2] Forn A, Baile MT, Rupérez E (2003). Spinel effect on the mechanical properties of metal matrix composite AA6061/(Al_2_O_3_)p. *Journal of Materials Processing Technology*.

[3] Lucey T, Wuhrer R, Yeung WY (2008). A quadrat analysis on particle distribution of cold rolled SiC_p_/Zn-22 wt%Al composites. *Materials Forum*.

[4] Ismail Ö, Ümit C, Kazim Ö (2000). The effect of forging on the properties of particulate-SiC-reinforced aluminium-alloy composites. *Composites Science and Technology*.

[5] Ochi Y, Masaki K, Matsumura T, Wadasako M (2004). Fatigue property and fatigue crack propagation behavior of Al_2_O_3_/A6061 MMCs at room and elevated temperature. *Key Engineering Materials*.

[6] Llorca J (2002). Fatigue of particle-and whisker-reinforced metal-matrix composites. *Progress in Materials Science*.

[7] Chawla N, Williams JJ, Saha R (2002). Mechanical behavior and microstructure characterization of sinter-forged SiC particle reinforced aluminum matrix composites. *Journal of Light Metals*.

[8] Long SG, Zhou YC (2005). Thermal fatigue of particle reinforced metal-matrix composite induced by laser heating and mechanical load. *Composites Science and Technology*.

[9] Ding H-Z, Biermann H, Hartmann O (2002). A low cycle fatigue model of a short-fibre reinforced 6061 aluminium alloy metal matrix composite. *Composites Science and Technology*.

[10] Heness GL, Ben-Nissan B, Gan LH, Mai Y-W (1999). Development of a finite element micromodel for metal matrix composites. *Computational Materials Science*.

[11] Fouret C, Degallaix S (2002). Experimental and numerical study of the low-cycle fatigue behaviour of a cast metal matrix composite Al-SiC_p_. *International Journal of Fatigue*.

[12] Oh KH, Han KS (2007). Short-fiber/particle hybrid reinforcement: effects on fracture toughness and fatigue crack growth of metal matrix composites. *Composites Science and Technology*.

[13] Hong S, Kim H, Huh D, Suryanarayana C, Chun BS (2003). Effect of clustering on the mechanical properties of SiC particulate-reinforced aluminum alloy 2024 metal matrix composites. *Materials Science and Engineering A*.

[14] Dan Z (1994). Effect of particle size on fracture toughness in metal matrix composites. *Engineering Fracture Mechanics*.

[15] Han NL, Yang J-M, Wang ZG (2000). Role of real matrix strain in low cycle fatigue life of a SiC particulate reinforced aluminum composite. *Scripta Materialia*.

[16] Lee JC, Subramanian KN, Kim Y (1994). The interface in Al_2_O_3_ particulate-reinforced aluminium alloy composite and its role on the tensile properties. *Journal of Materials Science*.

[17] Lloyd DJ (1994). Particle reinforced aluminium and magnesium matrix composites. *International Materials Reviews*.

[18] Iqbal AKMA, Arai Y, Araki W (2013). Effect of hybrid reinforcement on crack initiation and early propagation mechanisms in cast metal matrix composites during low cycle fatigue. *Materials & Design*.

